# Effectiveness and Safety of Cholecystectomy Versus Percutaneous Cholecystostomy for Acute Cholecystitis in Older and High-Risk Surgical Patients: A Systematic Review

**DOI:** 10.7759/cureus.70537

**Published:** 2024-09-30

**Authors:** Najeeb Ullah, Vaishnavi Kannan, Osman Ahmed, Sunitha Geddada, Amir T Ibrahiam, Zahraa M Al-Qassab, Iana Malasevskaia

**Affiliations:** 1 General Surgery, California Institute of Behavioral Neurosciences & Psychology, Fairfield, USA; 2 Internal Medicine, California Institute of Behavioral Neurosciences & Psychology, Fairfield, USA; 3 Research and Development, California Institute of Behavioral Neurosciences & Psychology, Fairfield, USA

**Keywords:** acute cholecystitis, cholecystectomy, cholecystostomy, comparative effectiveness, elderly, high-risk surgical patients, mortality rates, postoperative complications, safety, systematic review

## Abstract

Acute cholecystitis (AC) is a prevalent surgical emergency, particularly among elderly individuals who present with high perioperative risks. While early cholecystectomy (CCY) is the standard treatment, percutaneous cholecystostomy (PC) is proposed as an alternative for high-risk patients. This systematic review aims to evaluate the comparative safety and efficacy of CCY versus PC in managing AC among elderly and high-risk surgical patients. This review followed the Preferred Reporting Items for Systematic Reviews and Meta-Analyses (PRISMA) guidelines. A comprehensive search was conducted across multiple electronic databases, including PubMed/Medline, Cochrane Central Register of Controlled Trials (CENTRAL), ScienceDirect, Europe PMC, ClinicalTrials.gov, and EBSCO Open Dissertations, from July 1 to 15, 2024. Studies published from January 2019 to July 15, 2024, were included if they focused on patients aged 65 and older or those classified as high-risk surgical candidates. The review encompassed 72,366 participants across 22 studies, predominantly observational. Key outcomes assessed included postoperative complications, readmission rates, recurrence of cholecystitis, and mortality rates. This study highlights the need for individualized treatment strategies for managing AC in elderly populations. While CCY remains the preferred approach when feasible, PC offers a critical alternative for high-risk patients. Future research is necessary to optimize outcomes for this vulnerable population.

## Introduction and background

Acute cholecystitis (AC) is a common condition characterized by inflammation of the gallbladder, primarily caused by cystic duct obstruction from gallstones or impaired gallbladder function [1]. It presents a significant public health concern, affecting an estimated 20 million individuals annually in the United States [1]. Most cases, approximately 90%-95%, are attributed to cholelithiasis, with acalculous cholecystitis responsible for the remaining 5%-10% [2]. AC is the most frequent complication of cholelithiasis and one of the most common conditions necessitating emergency surgical intervention, particularly among the elderly population [3].

The incidence of cholelithiasis escalates with age, rendering the disease four to ten times more prevalent in older adults [4]. Cholelithiasis affects up to 30% of individuals over 60 [5]. Elderly patients are particularly susceptible to severe episodes of AC, with studies indicating that up to 6% of this demographic may experience significant complications [6]. Cholecystectomy (CCY) remains the most effective treatment for AC, with lower long-term biliary complications, shorter total admission length of stay, and lower overall treatment costs [7,8]. The UK's National Institute of Clinical Excellence (NICE) guidelines and the Tokyo Guidelines recommend it either early (within one to three days of admission) or as a delayed definitive procedure [9,10]. 

However, the benefits of an emergency cholecystectomy must be weighed against the higher risk of perioperative morbidity and mortality, making the management of elderly patients with acute cholecystitis a complex challenge [11]. A retrospective study from the UK found that patients over 80 who underwent laparoscopic cholecystectomy (LC) for AC had a 30-day mortality rate of 11.6% and a one-year mortality rate of 20.8% [12]. 

To address these concerns, some studies advocate for percutaneous cholecystostomy (PC) as a less invasive alternative for severely ill patients [13, 14]. This technique establishes a drainage pathway for bile but is considered a temporary solution, often leading to a high recurrence rate of cholecystitis [15, 16]. While PC may provide immediate relief, its long-term effectiveness compared to CCY is still being determined [17]. 

Given that elderly patients with multiple comorbidities are at an elevated risk for surgical complications, the optimal clinical management strategy remains ambiguous. While some experts recommend performing CCY only in patients who develop further complications, others suggest routine CCY following an initial PC [18].

This systematic review compares the effectiveness and safety of CCY versus PC in managing AC, specifically in elderly patients (65+) and those classified as high-risk surgical candidates. By providing a comprehensive analysis, this review seeks to guide clinical decision-making for managing AC in this vulnerable population.

## Review

Methods

This systematic review follows the Preferred Reporting Items for Systematic Reviews and Meta-Analyses (PRISMA) 2020 guidelines [19].

Search Strategy

A comprehensive search was conducted across multiple recognized electronic databases (PubMed/Medline, Cochrane Central Register of Controlled Trials (CENTRAL), ScienceDirect, Europe PMC, ClinicalTrials.gov, and EBSCO Open Dissertations) from July 1 to 15, 2024, to identify the relevant articles published between January 2019 and July 15, 2024. The search strategy employed a combination of Medical Subject Headings (MeSH) terms and 28 keywords pertinent to the research topic. This strategy was then adapted to meet each database's specific search syntax requirements (Table 1).

**Table 1:** Search Strategy MeSH: Medical Subject Heading, PMC: Pubmed Central

Search Base	Databases/Registers	Number of studies before/after the filters	Filters applied
((Aged OR "Older Adults") OR ("High-Risk Surgical Patients" OR "Comorbidity") OR "Aged[Mesh]) AND ( ‘’Cholecystitis, acute’’ OR ‘’Acute cholecystitis’’ OR ‘’acute calculous cholecystitis’’ OR ‘’Acute acalculous cholecystitis’’ OR cholecystitis OR ‘’Inflammation gallbladder’’ OR "Cholecystitis, Acute"[Mesh] "Cholecystitis, Acute/complications"[Mesh] OR "Cholecystitis, Acute/mortality"[Mesh] OR "Cholecystitis, Acute/surgery"[Mesh]) AND ((''Percutaneous Cholecystostomy'' OR "Cholecystostomy"[Mesh]) OR ("Emergency Cholecystectomy" OR ‘’urgent cholecystectomy’’ OR "Cholecystectomy"[Mesh])) AND (Readmission OR recurrence OR morbidity OR complications OR ‘’30-day mortality’’ OR (("Recurrence" [Me]) OR "Patient Readmission[Mesh]) OR "Hospital Mortality[Mesh]) OR "Morbidity"[Majr] OR "complications" [Subheading:NoExp])	PubMed/Medline	748/15	Full text, Clinical Study, Clinical Trial, Equivalence Trial, Observational Study, Randomized Controlled Trial, Humans, Aged: 65+ years, 2019-2024
#1 (Aged OR "Older Adults") OR ("High Risk Surgical Patients" OR "Comorbidity") 678416 #2 MeSH descriptor: [Frail Elderly] this term, only 1140 #3 #1 OR #2 678418 #4 (Cholecystitis,acute OR Acute cholecystitis OR acute acalculous cholecystitis OR Acute acalculous cholecystitis OR cholecystitis OR Inflammation of gallbladder OR Chronic cholecystitis):ti,ab,kw (Word variations have been searched) 2264 #5 MeSH descriptor: [Cholecystitis] this term only 332 #6 #4 OR #5 2264 #7 ‘’Laparoscopic cholecystectomy OR open cholecystectomy 6118 #8 MeSH descriptor: [Cholecystectomy, Laparoscopic] explode all trees 1769 #9 Cholecystostomy OR ‘’Gallbladder drain’’ OR ‘’gallbladder tube’’ OR ‘’Transhepatic gallbladder drain’’ OR ‘’Transhepatic gallbladder tube’’ OR ‘’cholecystostomy tube’’ 298 #10 MeSH descriptor: [Cholecystostomy] explode all trees 20 #11 #7 OR #8 OR #9 OR #10 6281 #12 Readmission OR recurrence OR morbidity OR complications 305564 #13 MeSH descriptor: [Postoperative Complications] explode all trees 55500 #14 #12 OR #13 329929 #15 #3 AND #6 AND #11 AND #14 310	Cochrane Library (Central)	310/99	Language: English Time Frame: Last 5 Years Studies Included: Clinical trials Studies Excluded: Reviews, Protocols, and Clinical Answers
((“Elderly” OR "older adults") AND ("Acute cholecystitis") AND ‘’laparoscopic cholecystectomy’’OR "open cholecystectomy" OR (“Cholecystostomy”) AND ("Readmission" OR "morbidity”)) AND (HAS_FT:Y) AND (((SRC:MED OR SRC:PMC OR SRC:AGR OR SRC:CBA) NOT (PUB_TYPE:"Review"))) AND (FIRST_PDATE:[2019 TO 2024])	Europe PMC	310/107	Study design: Full text, research articles Time Frame: 5 years
(“Elderly” OR "older adults" OR High risk patients") AND ("Acute cholecystitis" ) AND (‘’laparoscopic cholecystectomy’’OR "open cholecystectomy" OR Cholecystostomy) AND ("Readmission" OR "morbidity")	Science Direct	2460/59	Study Design: Research Articles' Subject area: medicine and dentistry Time Frame: five years Open-access articles only
(“Elderly” OR "older adults" OR High risk patients") AND ("Acute cholecystitis" ) AND (‘’laparoscopic cholecystectomy’’OR "open cholecystectomy" OR Cholecystostomy) AND ("Readmission" OR "morbidity") AND ( RCT OR cohort OR ''case-control'' OR ''clinical study'' OR '' Randomized Trial'')NOT(''Review'' OR ''Meta-analysis'')	EBSCO Open Dissertations	1383/484	Publication Date: 20190101-20241231 Language: English
Condition/disease: ("Acute cholecystitis" ) OR (‘’laparoscopic cholecystectomy’’OR "open cholecystectomy" OR Cholecystostomy) Intervention/treatment :(‘’laparoscopic cholecystectomy’’OR "open cholecystectomy" OR cholecystostomy)	ClinicalTrials.gov	20/0	Age 65+

Inclusion and Exclusion Criteria

Studies were included if they met the eligibility criteria, including patient age (65+), high-risk surgical status, study design, interventions involving cholecystectomy (CCY) or cholecystostomy, and outcomes such as postoperative complications, readmission, recurrence, morbidity, and 30-day mortality. Only studies published in English and conducted within the last five years were considered. Eligibility criteria are detailed below in Table 2.

**Table 2:** Inclusion and Exclusion Criteria CCY: Cholecystectomy; AC: acute cholecystitis; PC: percutaneous cholecystostomy; RCT: randomized controlled trial; NRCTs: non-randomized clinical trials

Criteria	Inclusion	Exclusion
Population	Patients with acute cholecystitis (AC), age more than 65 years, high-risk surgical patients, and comorbidities	Patients with chronic cholecystitis, acute pancreatitis, cholangitis, and choledocholithiasis aged less than 65 years
Intervention	CCY or PC	Interventions other than CCY or PC
Outcome	Postoperative complications, readmission, recurrence, morbidity, and 30-day mortality	Studies do not focus on complications, readmission, recurrence, morbidity, and 30-day mortality.
Study Design	RCTs, high-quality observational studies (e.g., retrospective, prospective cohort, case-control), and NRCTs	Case reports, case series, systematic reviews, meta-analyses, and expert opinions
Year of publication	Studies published from January 2019 to July 15, 2024	Studies published before January 2019 or after July 15, 2024
Language	Published in English	Other languages

Screening and Quality Assessment

A rigorous two-stage screening process was employed, utilising the Rayyan app® (Ouzzani et al., 2016) [20] for initial record management. Initially, a single reviewer screened titles and abstracts to find potentially pertinent studies, followed by two independent reviewers screening all the titles and abstracts for eligibility. Disagreements were resolved through discussion or by consulting a third reviewer. We employed the Newcastle-Ottawa Scale (NOS) [21] assessment for observational studies to evaluate the quality of the studies included in this review.

Data Extraction

A standardized data extraction form was created to gather relevant information from the selected studies. This information encompassed critical aspects of the studies, such as study characteristics, intervention details, outcome measures, and necessary results. Two independent reviewers performed data extraction. Any discrepancies were resolved through discussion or consultation with a third reviewer.

Data Synthesis

The extracted data was then synthesized using the design and outcome measures of the included studies. Due to the anticipated heterogeneity in study designs and methodologies, a narrative synthesis approach was adopted.

Results

**Figure 1:**
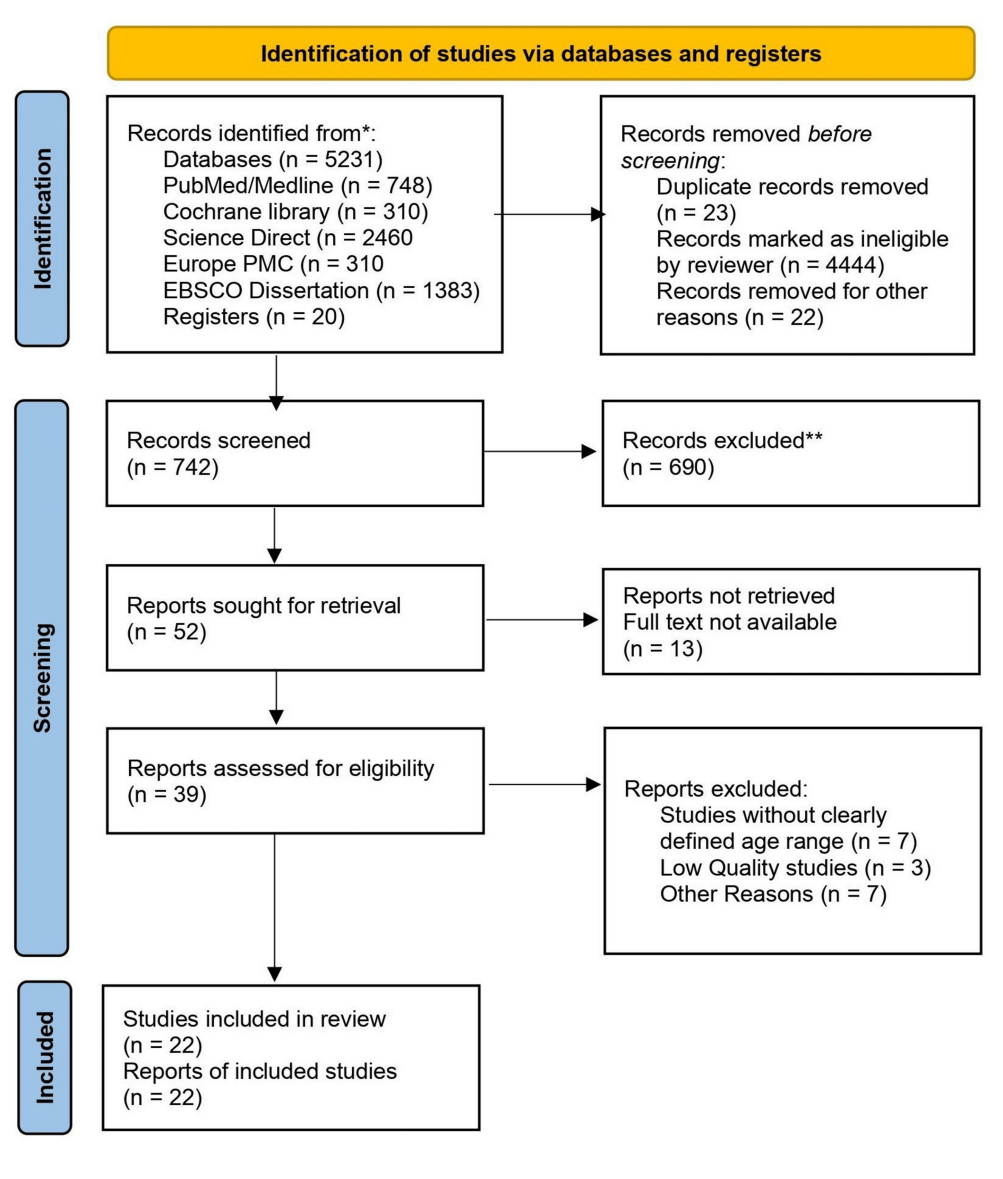
PRISMA 2020 flowchart depicting the process for the article selection *Consider, if feasible to do so, reporting the number of records identified from each database or register searched (rather than the total number across all databases/registers). **If automation tools were used, indicate how many records were excluded by a human and how many were excluded by automation tools. PRISMA: Preferred Reporting Items for Systemic Reviews and Meta-Analyses; PMC: PubMed Central

The initial database search yielded 5231 results. After applying the pre-defined inclusion and exclusion criteria detailed in Table 2, 742 studies were deemed suitable for screening. From this pool, 52 studies were selected for retrieval, and 39 were subsequently assessed for eligibility. Ultimately, 22 studies met all criteria for inclusion in the review, as illustrated in Figure 1.

Risk of Bias Assessment

We employed the Newcastle-Ottawa Scale (NOS) [21] assessment for observational studies to evaluate the quality of the studies included in this review. Two independent reviewers conducted the assessment, and any discrepancies in their evaluations were resolved through consensus or arbitration.

The NOS focuses on three critical domains: the selection of study groups, the comparability between the study and control groups, and the assessment of study outcomes. Each study was assigned a score ranging from zero to nine points, allowing for a systematic quality evaluation. Studies were categorized as having good, fair, or poor quality based on the overall risk of bias assessment. Specifically, studies that scored at least seven out of nine points (equivalent to 77%) were considered acceptable for inclusion in the review.

Three of the 25 observational studies assessed were classified as poor quality and were subsequently excluded from further analysis. This exclusion was necessary to ensure that the conclusions drawn from the review were robust and less biased, enhancing the overall validity of the findings (see Table 3).

**Table 3:** Quality Assessment of Observational Studies: Newcastle-Ottawa Scale Scores Note: We could award a maximum of four stars (*) for selection, two for comparability, and three for outcome domains. The total score ranges from zero to nine

Study/ Year	Selection	Comparability	Outcome	Overall
Hess et al., 2021 [22]	****	**	***	9/Good Quality
Jeon et al., 2020 [23]	****	**	***	9/Good Quality
Ramírez-Giraldo et al., 2024 [24]]	****	*	***	8/Good Quality
Curry et al., 2024 [25]	****	*	***	8/Good Quality
Cruz-Centeno et al., 2022 [26]	****	*	***	8/Good Quality
Bhatia et al., 2023 [27]	***	-	***	6/Poor Quality
Köstenbauer et al., 2023 [28]	***	*	***	7/Good Quality
Sgantzou et al., 2022 [29]	***	*	***	7/Good Quality
Kang et al., 2022 [30]	***	*	***	7/Good Quality
Park et al., 2022 [31]	***	*	***	7/Good Quality
Er et al., 2020 [32]	**	*	***	6/Poor Quality
Yao et al., 2021 [33]	**	*	***	6/Poor Quality
Søreide et al., 2020 [34]	***	**	***	8/Good Quality
Escartin et al., 2019 [35]	***	**	***	8/Good Quality
Fagenson et al., 2020 [36]	***	**	***	8/Good Quality
Dvorak et al., 2019 [37]	***	**	***	8/Good Quality
Rubio-García et al., 2023 [38]	***	**	***	8/Good Quality
Roesch-Dietlen et al., 2019 [39]	****	**	***	9/Good Quality
Nishida et al., 2019 [40]	****	**	***	9/Good Quality
Chen et al., 2021 [41]	****	**	***	9/Good Quality
Ramírez‑Giraldo et al., 2023 [42]	****	**	***	9/Good Quality
Yirgin et al., 2023 [43]	****	**	***	9/Good Quality
Bass et al., 2021 [44]	****	**	***	9/Good Quality
Doğrul et al., 2022 [45]	****	**	***	9/Good Quality
González-Castillo et al., 2021 [46]	****	**	***	9/Good Quality

Characteristics of the included studies

Our systematic review encompasses 22 observational studies, primarily retrospective cohort studies, conducted across various countries, including Switzerland, the United States, the United Kingdom, Colombia, Australia, Greece, Turkey, China, Spain, Japan, Norway, the Czech Republic, and Mexico. The sample sizes ranged from a minimum of 44 to a maximum of 47,478 patients, with a total of 72,366 participants, with most studies focusing on elderly populations, often with mean ages around 70-85 years. The key outcomes of interest included the effectiveness of percutaneous cholecystostomy (PC) as a bridge to laparoscopic cholecystectomy (LC) or early cholecystectomy in high-risk elderly patients, as well as the morbidity and mortality associated with PC and cholecystectomy (CCY). Additional outcomes examined were the recurrence of acute cholecystitis (AC), length of hospital stay, and survival rate (Table 4).

**Table 4:** Summary of the Included Studies AC: Acute cholecystitis; AAC: acute acalculous cholecystitis; ACC: acute calculous cholecystitis; ACCBD: acute complicated calculous biliary disease; PCT: percutaneous cholecystostomy tube; CCY: cholecystectomy; EC: early cholecystectomy; DLC: delayed laparoscopic cholecystectomy; ASA: American Society of Anesthesiologists; CCI: Charlson Comorbidity Index; NSW: New South Wales; ACS: American College of Surgeons; ICU: intensive care unit; LOS: length of stay; HPBP: hepatopancreatobiliary pathology; GPC: gallbladder-preserving cholecystolithotomy; CHD: coronary heart disease; CHF: congestive heart failure; QoL: quality of life; COPD: chronic obstructive pulmonary disease; TG: Tokyo Guidelines; PTGBD, PTGD: percutaneous transhepatic gallbladder drainage

Author/Year	Type of study	Country	Sample size	Mean Age (years ± SD) / Median Age (years)	Aim of study	Key findings
Hess et al., 2021 [22]	Retrospective cohort study	Switzerland	158	76.5	This study provided a detailed analysis of patients treated with PC over a prolonged period, focusing on their health condition, clinical outcomes, and the timing of secondary surgery.	50% of patients received PC with CCY, resulting in an 85% decrease in mortality. In patients who had PC alone without CCY, 48% (38 patients) died, compared to 9% with PC and subsequent CCY. PC remains a temporary measure; upfront, CCY was proposed.
Jeon et al., 2020 [23]	Retrospective cohort study	Korea	196	66.97 ± 15.231 (ELC after PTGBD), 64.97 ± 12.978 (DLC after PTGBD)	This study aimed to ascertain the optimal timing for surgical intervention in Grade II acute cholecystitis patients who had PTGBD insertion.	Patients treated with ELC showed a significantly longer mean operative time (P = 0.001) and longer postoperative hospital stay (P = 0.014) than those treated with DLC. DLC was found to produce better outcomes than ELC.
Ramírez-Giraldo et al., 2024 [24]	Retrospective cohort study	Colombia	144	90 (Median age as reported in the study)	The study aimed to identify the factors influencing two-year survival following laparoscopic cholecystectomy in patients over 80.	37 patients (25.69%) died during the two-year follow-up. The two-year survival rate for ASA 1-2 patients was 87.50%, compared to 63.75% (p=0.001) for ASA 3-4 patients. An ASA score of 3–4 was a statistically significant predictor of mortality (HR: 2.71, 95% CI: 1.20–6.14).
Curry et al., 2024 [25]	Retrospective cohort study	United States	13,782	71.1 ± 13.1 vs 67.4 ± 15.3	The study aimed to assess the outcomes of delayed CCY in patients with grade III acute cholecystitis who were deemed to be at high peri-operative risk upon index admission and received PCT versus those who were not.	13.3% of the patients underwent PCT. PCT patients were older and had a lower risk of respiratory (AOR 0.67, CI 0.54-0.83) and infectious complications (AOR 0.77, CI 0.62-0.96) after CCY. PCT exhibited comparable pLOS (β +0.31, CI [-0.14, 0.77]) and operative hospitalization costs (β $800, CI [− 2300, +600]). PCT may effectively bridge patients with grade III acute cholecystitis to CCY.
Cruz-Centeno et al., 2022 [26]	Retrospective cohort study	San Juan, Puerto	95	79 (median age as reported in the study)	The study compared the outcomes of performing an interval cholecystectomy versus conservative management following PCT placement in AC patients.	26.3% of the patients underwent an interval cholecystectomy. The surgical group had a higher rate of readmissions and biliary complications. Choledocholithiasis (44% vs. 21.4%; p = 0.03) necessitates prompt cholecystectomy in PCT patients.
Köstenbauer et al., 2023 [28]	Retrospective cohort study	Australia	47,478	66 ± 10.2 (early surgery), 6 ± 10.7 (delayed surgery)	The study compared health outcomes and factors influencing variation, as well as reports the proportion of early versus DC in older patients in NSW (Australia),	Within seven days of admission, 85% of older patients underwent CCY. Early surgery was linked to reduced rates of bile duct injury (0.18%), readmissions, conversion to open surgery, and overall length of stay (p < 0.001).
Sgantzou et al., 2022 [29]	Retrospective cohort study	Greece	86	72.2	The study aimed to ascertain the mortality and morbidity of PC performed under CT guidance in patients at high surgical risk.	Patients who were admitted to the ICU had significantly higher 7- and 30-day mortality rates (16.3% and 22.1%, respectively) (P<0.05). There was no statistically significant relationship between age (P = 0.563), gender (P = 0.186), catheter diameter (P = 0.932), diagnosis (P = 0.354), or length of hospital stay (P = 0.426). In patients with high perioperative risk, PC was a safe alternative to surgery with acceptable mortality rates.
Kang et al., 2022 [30]	Retrospective cohort study	China	74	73 + 6.3	The study assessed the value of combining PTGD with GPC in high-risk patients with ACC.	59 patients underwent laparoscopic cholecystectomy, with four having complications (6.8%). In total, 14 patients received GPC. PTGD, in combination with GPC, was a safe and effective treatment for high-risk patients with acute calculous cholecystitis who could not receive DC.
Park et al., 2022 [31]	Prospective cohort study	Korea	95	Non-PTGBD group 58.9±14.4, PTGBD 69.9±11.4	This study compared the QoL of PTGBD patients and non-PTGBD patients who participated in a prospective study evaluating complication monitoring and validation.	69 non-PTGBD and 21 PTGBD patients. The PTGBD group consisted of older and more morbid patients. The PTGBD group experienced longer operation times (72.4±34.7 vs. 52.8±22.0 minutes, P=.022) and had a significantly higher complication rate (38.1% vs. 10.1%, P=.003). There was no significant difference in the global health scale before or after surgery, but the functional and emotional scales were better in the PTGBD groups.
Søreide et al., 2020 [34]	Retrospective cohort study	Norway	149	72.5	This study aimed to assess the demographics and outcomes of PC in the treatment algorithm for routine AC clinical practice using a population-based cohort.	PC is beneficial to certain AC patients. Only 50% of the patients received definitive surgical treatment. Improved decision-making based on disease severity, comorbidities, and available treatment options may result in more tailored care and less use of non-definitive treatments.
Escartín et al., 2019 [35]	Retrospective cohort study	Spain	348	85.4	This study assessed the characteristics, management, and outcomes of acute cholecystitis in patients ≥ 80 years old.	Treatment options for elderly patients with AC are determined by disease severity (grade III AC) and physical status (ASA III-IV) rather than age alone. Laparoscopic cholecystectomy was safe and effective for grade I –II AC, even in elderly patients. Grade III AC patients have a high risk of morbidity and mortality; treatment should be tailored to the individual. ASA IV patients should avoid cholecystectomy; antibiotic treatment and cholecystectomy are preferable
Fagenson et al., 2020 [36]	Prospective cohort study	United States	6898	-	The study aimed to determine the relationship between frailty, postoperative morbidity, and mortality in patients undergoing laparoscopic cholecystectomy for acute cholecystitis.	Clavien IV complications were more common in intermediate frail patients (OR 1.81; p = 0.050) and high-frail patients (OR 4.59; p < 0.001). Mortality rates were also higher for intermediate frailty (OR 4.69; p = 0.014) and high frailty (OR 12.2; p = 0.001). Frailty is associated with increased postoperative morbidity and mortality in laparoscopic cholecystectomy for acute cholecystitis.
Dvorak et al., 2019 [37]	Retrospective cohort study	Czech Republic	69	78.5	The study aimed to assess the indications, technical aspects, efficacy, complications, and patient outcomes of percutaneous CT-guided cholecystectomies for AC and the procedure's role in treatment selection.	Sepsis was an indication for intervention in 34 cases (45.3%). Fifteen patients (20%) experienced acute gallbladder inflammation, while eight patients (10.7%) had severe medical conditions. PC was the most frequently used treatment for acalculous cholecystitis (79.3%). The 30-day mortality rate was 10.7%, while the overall complication rate was 21.3%. PC was frequently used as the final treatment for acalculous cholecystitis (79.3%). The 30-day mortality rate was 10.7%, with a total complication rate of 21.3%.
Rubio‑García et al., 2023 [38]	Retrospective cohort study	Spain	195	74.64	This study evaluated the indications, patients’ characteristics, and morbidity and mortality in patients with ACC treated with PC and patients who undergo cholecystectomy following PC.	The complication rate for PC is 12.3%, with a 90-day mortality rate of 14.4%. The average duration of PC use was 10.7 days, with emergency surgery required in 4.6%. PC had an overall success rate of 66.7%, a one-year readmission rate for biliary complications of 28.2%, and a scheduled cholecystectomy rate of 22.6%. Although PC effectively reduces inflammation and infection in AC, patient age, morbidity, and comorbidity scores all contribute to high mortality.
Roesch-Dietlen et al., 2019 [39]	Retrospective cohort study	Mexico	223	48.8 ± 23.45 group A and 49.2 ± 13.47, group B	The study aimed to ascertain the safety of laparoscopic subtotal cholecystectomy in patients with acute cholecystitis.	Total cholecystectomy (82.95%) and subtotal cholecystectomy (17.05%) were performed. The results were not statistically significant between the two groups. Subtotal cholecystectomy was a helpful treatment option for patients with acute cholecystitis. It was a safe and reliable method of preventing bile duct injury.
Nishida et al., 2019 [40]	Retrospective cohort study	Japan	182	92.5	The study aimed to investigate the postoperative outcomes of elderly patients with and without dementia who underwent early cholecystectomy for AC.	The overall complication rate after early cholecystectomy for AC in 59 patients was 11%. There was no mortality in this series. The regular discharge rate was 89.8%, and the median (LOS) was 9.0 days. Out of the 59 patients, 22 (37.3%) had a history of dementia. Complication rates were comparable between the groups, even though the dementia group developed delirium at a significantly higher rate. EC is safe for older people with dementia.
Chen et al., 2021 [41]	Retrospective cohort study	China	44	73.5	The study aimed to identify predictors of patient outcomes when PC is chosen as the final treatment option and risk factors for relapse in moderate and severe AAC patients following initial PC.	During the 17-month follow-up period following catheter removal, 21 patients (47.7%) did not have cholecystitis recurrence, 6 (13.6%) did, and 17 (38.6%) died (5–60 days). Multivariate analysis revealed that CHD and CCF (OR 26.50; P = 0.038) were associated with recurrence. The age-adjusted CCI (OR 1.53; P = 0.018) had an independent association with 60-day mortality after PC.
Ramírez‑Giraldo et al., 2023 [42]	Retrospective cohort study	Colombia	600	65.0	The study aimed to examine the safety and outcomes of laparoscopic cholecystectomy in patients older than 90 years.	Nonagenarians had higher rates of complications, conversion, subtotal cholecystectomy, and longer hospitalizations. Overall mortality was 1.6%, with 6.8% among those aged 90 and older. Regression models identified significant risk factors for mortality: age over 90 years (RR 4.6), presence of cholecystitis (RR 8.2), and time from admission to cholecystectomy (RR 1.2).
Yirgin et al., 2023 [43]	Retrospective cohort study	Türkiye	122	66.4±17.8	The study investigated the impact of timing on the clinical process of high-risk AC patients undergoing PC. The median follow-up period was 26.6 months.	In total, 98 patients (80.3%) underwent early PC, while 24 patients (19.7%) underwent late PC (>72 hours). The median LOS was six days in the early PC group and nine days in the late PC group (P<0.001). The mean age, HPBP, and surgery rate did not differ significantly between the early and late PC groups.
Bass et al., 2021 [44]	Retrospective cohort study	United States	338	79 ± 8	The study aimed to record the prevalence of ACCBD in elderly patients in Europe and the current treatment regimen.	Only 37.8% of patients over 65 underwent surgery compared to 64.7% (p < 0.001). Surgical complications and mean postoperative LOS were higher in the over-65 group. Postoperative mortality was 2.2% in people over 65 (vs. 0.7%; p = 0.253). Elderly patients had higher morbidity and LOS than younger patients, but they did not differ from elderly patients treated non-operatively.
Doğrul et al., 2022 [45]	Retrospective cohort study	Türkiye	127	69±13.5	The study aimed to determine which factors can be considered when making interval CCY decisions and to forecast death in high-risk AC patients.	43.1% of patients underwent an elective cholecystectomy. The mortality rates after 30 days and a year were 11% and 17.3%, respectively. ASA score, CCI, the negative predictive factors described in the Tokyo Guidelines 2018, and the ACS expected mortality rate and albumin level were discovered to be significant predictors of mortality and elective CCY probability. PC is sufficient for resolving inflammation; however, medical comorbidities determine patients' final condition.
González-Castillo et al., 2021 [46]	Retrospective cohort study	Spain	963	66.5	The study aimed to identify and evaluate mortality risk factors in ACC using the TG classification.	The overall mortality rate was 3.6%, with older age (68 + IQR 27 vs. 83 + IQR 5.5; P = 0.001) and higher CCI (3.5 + 5.3 vs. 0 + 2; P = 0.001). COPD (OR 4.66, 95% CI 1.7-12.8, P = 0.001), dementia (OR 4.12, 95% CI 1.34-12.7, P = 0.001), and age over 80 (OR 1.12, 95% CI 1.02-1.21, P = 0.001) all predicted death in more than 90% of patients. Mortality was higher in the nonsurgical treatment group. (26.2% vs. 10.5%)

Discussion

This discussion summarizes findings from observational studies conducted in various countries. The studies focused on AC patients and the management strategies used, particularly PC and early or delayed CCY. The included studies consistently show a trend toward better outcomes when surgical interventions are timed and tailored to the individual patient's health status, particularly in elderly or high-risk populations.

Efficacy of Percutaneous Cholecystostomy

The studies strongly support the use of PC as an early critical intervention for patients with AC, particularly those who are at high surgical risk. Hess et al. (2021) illustrated that patients who underwent PC with subsequent CCY had an 85% lower mortality risk [22]. Additionally, Curry et al. (2024) noted that patients receiving PC exhibited reduced odds of respiratory and infectious complications after eventual CCY [25]. PC effectively bridges definitive surgical management and facilitates recovery by addressing acute inflammation and infection, as highlighted by Doğrul et al. (2022) [45]. Furthermore, Chen et al. (2021) reported low recurrence rates of cholecystitis in patients who had PC as their final treatment option [41].

Yirgin et al. (2023) emphasized the direct benefits of early PC, noting that timely intervention resulted in shorter hospital stays, highlighting the importance of prompt intervention in improving patient outcomes [43]. Furthermore, Sgantzou et al. (2022) [29], Dvorak et al. (2019) [37], and Roesch-Dietlen et al. [38] documented low complication and mortality rates associated with PC, affirming its role in managing severe AC cases.

Mortality Risk Factors and Age Considerations

In these studies, a recurring theme is the correlation between age, comorbidities, and increased mortality rates associated with AC treatment. Ramírez-Giraldo et al. (2024) found that patients with higher American Society of Anesthesiologists (ASA) scores had increased mortality rates at the two-year follow-up after laparoscopic CCY, highlighting the influence of overall health status on surgical outcomes [24]. Factors such as dementia, chronic obstructive pulmonary disease (COPD), a higher CCI, and advanced age were identified as significant mortality predictors by González-Castillo et al. (2021) [46]. Fagenson et al. (2020) found that high-frail patients undergoing laparoscopic cholecystectomy for acute cholecystitis were more likely to experience Clavien IV complications, increased postoperative morbidity, and mortality [36].

The Escartín et al. (2019) study emphasizes the need to evaluate surgical options on a case-by-case basis, taking into account older individuals' physical status (ASA score) and disease severity [35]. This highlights the importance of making personalized treatment decisions based on overall health rather than chronological age.

Timing of Surgery and Resource Utilization

Cost-effectiveness and resource utilization are another area of concern. Köstenbauer et al. (2023) highlighted that early cholecystectomies provide favorable postoperative outcomes such as reduced length of stay, fewer readmissions, and lower conversion rates to open surgery [28]. This concept is based on the premise that timely intervention improves clinical outcomes and may result in more efficient use of healthcare resources.

On the other hand, studies by Doğrul et al. (2022) [45] and Søreide et al. (2020) [34] support interval cholecystectomies based on comorbid profiles and clinical stability, emphasizing the need for careful timing in choosing between conservative management and surgical intervention post-PC. Cruz-Centeno et al. (2022) stress the significance of subsequent CCY in AC patients receiving antibiotics or PC to prevent biliary complications, particularly choledocholithiasis [26].

Additionally, Nishida et al. (2019) emphasized that early cholecystectomy is safe for elderly patients with dementia [40]. In contrast, Jeon et al. (2020) demonstrated that delayed laparoscopic cholecystectomy (DLC) produces better outcomes than early laparoscopic cholecystectomy (ELC) [23]. According to Ramírez-Giraldo et al. (2023), age, the presence of cholecystitis, and the time from admission to surgery, all affect how safe laparoscopic cholecystectomy is for the elderly [42].

The findings highlight the importance of personalized treatment approaches, especially for elderly patients with comorbidities. Higher ASA scores and conditions such as dementia and COPD are significant predictors of mortality, emphasizing the importance of tailored surgical options based on individual health profiles rather than chronological age.

This review highlights the necessity for a multidisciplinary approach to managing AC, advocating for individualized treatment plans for each patient's unique health status. Future research should prioritize randomized controlled trials and longitudinal studies to further clarify these interventions' long-term outcomes and cost-effectiveness, ultimately enhancing management strategies for this vulnerable population.

Implications for Clinical Practice

The collective insights from these studies advocate for a paradigm shift in managing acute cholecystitis, particularly among the geriatric and high-risk cohorts. There is a clear message regarding the benefits of PC as a safe, minimally invasive option that paves the way for definitive surgical interventions. Clinicians must adopt a multidisciplinary approach, utilizing a patient's comorbidity profile, frailty status, and overall health when designing treatment plans.

Further research will be critical for refining PC indications, understanding long-term outcomes post-surgery, and determining the optimal timing for surgical interventions to minimize complications and enhance patient recovery.

Comparison with Other Evidence 

When comparing our findings with existing systematic reviews on the management of AC, several similarities and differences emerge, accompanied by potential explanations for these observations. A notable similarity is the effectiveness of PC, which Cirocchi et al. (2023) advocate as a viable intervention for high-risk patients [47]. Our review and Cirocchi et al.'s work underscore the role of PC as a critical bridge to definitive surgical management, particularly for patients unable to undergo immediate surgery [47]. In contrast, the systematic reviews by Gurusamy et al. (2010) [48] and Cirocchi et al. (2023) [49 ] assert that early cholecystectomy (EC) should be regarded as the treatment of choice, even among high-risk surgical patients. Another area of consensus is the significance of timely intervention. Numerous studies agree that prompt surgical intervention, whether through early cholecystectomy or timely percutaneous cholecystostomy, yields improved patient outcomes. This is consistent with findings from Köstenbauer et al. (2023), which demonstrate that early interventions are associated with reduced hospital stays and lower complication rates [28]. Furthermore, the relationship between age, comorbidities, and elevated mortality rates emerges as a recurring theme in our review and the existing literature. Studies consistently identify similar risk factors, including higher ASA scores and conditions such as COPD and dementia, as significant predictors of adverse outcomes.

However, differences also arise, particularly concerning the comparative effectiveness of PC versus early CCY. While our review supports the efficacy of PC as a bridge to surgery, some systematic reviews, such as those by Markopoulos et al. (2021) [50], suggest that patients managed with PC may experience higher mortality and readmission rates compared to those who undergo early CCY. This discrepancy may stem from differences in study populations, definitions of high-risk patients, or variations in surgical protocols. Furthermore, our review emphasizes the need for personalized timing based on clinical stability, whereas some systematic reviews advocate for early CCY as the preferred approach, even in high-risk cases. This discrepancy may result from different interpretations of "early" intervention and the criteria used to assess clinical stability.

Long-term outcomes also show a point of divergence. Many systematic reviews highlight short-term outcomes, while our review calls for a greater focus on long-term follow-ups to assess recurrence rates and late complications. This indicates a gap in the existing literature regarding the durability of treatment outcomes, particularly in elderly populations.

Several factors contribute to these differences. Variability in the study design, including heterogeneous populations or varying definitions of high-risk patients, can lead to divergent findings. For instance, some reviews may encompass a broader range of surgical candidates, while others focus specifically on the elderly or those with multiple comorbidities. Methodological variations, including sample sizes and statistical analyses, can also affect the robustness of conclusions in different systematic reviews. Moreover, the reliance on published studies may introduce publication bias, as studies with negative or inconclusive results are less likely to be disseminated, potentially skewing the perceived effectiveness of particular interventions, like PC. Finally, the field of surgical management for AC is rapidly evolving, with ongoing advancements in techniques and technologies. As new evidence emerges, it may lead to shifts in clinical practice that are not yet reflected in all systematic reviews.

While our findings and those of existing systematic reviews regarding the management of AC share notable similarities, significant differences also exist, particularly concerning the comparative effectiveness of interventions and the timing of surgical procedures. Understanding these similarities and discrepancies is essential for refining treatment protocols and enhancing patient outcomes, especially in high-risk populations. Future research should aim to standardize methodologies and focus on long-term outcomes.

Strengths and Limitations of the Included Studies and the Review Process

The review presents several notable strengths and limitations concerning the comparative effectiveness of PC and CCY for AC in elderly and high-risk populations. A significant strength is the review's comprehensive search strategy, which employed a rigorous and systematic approach across multiple electronic databases. This methodology ensures a broad collection of relevant studies, addressing the critical question of effectiveness in the specified demographic. Furthermore, using the Newcastle-Ottawa Scale to assess the quality of observational studies lends considerable credibility to the findings. By excluding studies of lower quality, the review enhances the robustness and reliability of its conclusions. The review adds value by targeting vulnerable populations, particularly elderly and high-risk patients, a demographic often underrepresented in clinical research. The review provides essential insights into clinical outcomes relevant to managing AC by focusing on this group. Additionally, the diversity of the study population, with research conducted across various countries, contributes to the generalizability of the findings concerning different healthcare systems and practices.

However, the review has its limitations. One notable concern is the heterogeneity of the included study designs, which complicates direct comparisons. Variations in methodologies, sample sizes, outcome definitions, and statistical analyses must be more consistent in the findings. Additionally, publication bias may impact the review, as studies with positive outcomes are more likely to be published, whereas those with negative or inconclusive results may be underreported. The absence of randomized controlled studies (RCTs) and meta-analyses in our review, along with the reliance on observational studies, increases the potential for biases and confounding factors that may affect the accuracy and validity of the results. Furthermore, many studies in the review reported only short follow-up durations, which restricts the ability to evaluate long-term outcomes and the potential for late complications or recurrences following treatment.

Future Directions for Research

Several key directions are suggested for future research in this field. Longitudinal studies are emphasized to assess the durability of treatment outcomes, particularly concerning recurrence rates of cholecystitis and late complications that may arise following PC or CCY in elderly patients. Considering the difficulties of conducting RCTs in high-risk and elderly populations, future research should focus on using pragmatic trial designs and advanced observational studies. Pragmatic trials can better reflect real-world conditions and address some of the practical challenges of traditional RCTs, while well-designed observational studies with rigorous analytical methods can offer valuable complementary insights into the comparative effectiveness of cholecystectomy versus percutaneous cholecystostomy.

Furthermore, future studies should include economic evaluations to analyze the cost-effectiveness of both interventions, taking into account factors such as hospital stay duration, readmission rates, and long-term healthcare costs associated with each option. Finally, standardizing outcome measures and definitions in future research will enhance comparability across studies and facilitate more robust meta-analyses. Future research that addresses these areas can contribute to a more comprehensive understanding of AC management and improve care for vulnerable populations.

## Conclusions

This systematic review highlights the critical comparison between CCY and PC in managing AC among elderly and high-risk patients. While both interventions have their merits, CCY remains the primary surgical approach when feasible, as evidenced by lower mortality rates and fewer complications during the optimal timing of the intervention. Conversely, PC serves as a crucial bridge for the high-risk population, allowing for temporary alleviation of symptoms and stabilization before definitive surgical management. However, the review underscores the necessity for individualized treatment plans tailored to each patient's comorbidities, frailty, and overall health status. Future research should explore pragmatic trials that reflect real-world settings and advanced observational studies to provide complementary insights into the comparative effectiveness of CCY and PC.
